# Evaluation of chronic lymphocytic leukemia by BAC-based microarray analysis

**DOI:** 10.1186/1755-8166-4-4

**Published:** 2011-02-03

**Authors:** Roger A Schultz, Maria Delioukina, Karl Gaal, Victoria Bedell, David D Smith, Stephen J Forman, Lisa D McDaniel, Blake C Ballif, Lisa G Shaffer, Marilyn L Slovak

**Affiliations:** 1 Signature Genomics, 2820 N. Astor St., Spokane, WA, 99207, USA; 2 Department of Hematology/Hematopoietic Cell Transplantation, City of Hope, 1500 E. Duarte Rd., Duarte, CA, 91010, USA; 3 Department of Pathology, City of Hope, 1500 E. Duarte Rd., Duarte, CA, 91010, USA; 4 Department of Cytogenetics, City of Hope, 1500 E. Duarte Rd., Duarte, CA, 91010, USA; 5 Department of Biostatistics, City of Hope, 1500 E. Duarte Rd., Duarte, CA, 91010, USA; 6 Quest Diagnostics Nichols Institute, Chantilly, VA, USA

## Abstract

**Background:**

Chronic lymphocytic leukemia (CLL) is a highly variable disease with life expectancies ranging from months to decades. Cytogenetic findings play an integral role in defining the prognostic significance and treatment for individual patients.

**Results:**

We have evaluated 25 clinical cases from a tertiary cancer center that have an established diagnosis of CLL and for which there was prior cytogenetic and/or fluorescence *in situ *hybridization (FISH) data. We performed microarray-based comparative genomic hybridization (aCGH) using a bacterial artificial chromosome (BAC)-based microarray designed for the detection of known constitutional genetic syndromes. In 15 of the 25 cases, aCGH detected all copy number imbalances identified by prior cytogenetic and/or FISH studies. For the majority of those not detected, the aberrations were present at low levels of mosaicism. Furthermore, for 15 of the 25 cases, additional abnormalities were detected. Four of those cases had deletions that mapped to intervals implicated in inherited predisposition to CLL. For most cases, aCGH was able to detect abnormalities present in as few as 10% of cells. Although changes in ploidy are not easily discernable by aCGH, results for two cases illustrate the detection of additional copy gains and losses present within a mosaic tetraploid cell population.

**Conclusions:**

Our results illustrate the successful evaluation of CLL using a microarray optimized for the interrogation of inherited disorders and the identification of alterations with possible relevance to CLL susceptibility.

## Background

B-cell chronic lymphocytic leukemia (CLL) is the most common of the leukemias, accounting for ~30% of all cases [1]. CLL exhibits a highly variable course with life expectancies ranging from only a few months to many decades. Cytogenetic evaluation is a key component in the diagnosis of this disorder and, importantly, in defining the prognostic significance of the disease and optimal treatment choices for individual patients. The most common cytogenetic aberrations identified through conventional karyotyping and fluorescence *in situ *hybridization (FISH) are deletion 13q14, deletion 6q, trisomy 12, deletion 11q22q23 and deletion 17p13. More than 80% of patients show one or more of these alterations by chromosome analysis, with approximately the same number seen by FISH. These recurrent cytogenetic abnormalities have prognostic significance as they are associated with an increasingly poorer outcome in the order listed [2-4]. Thus, deletion of 13q14, which is the most common finding and is seen in 57% of cases analyzed by FISH and 55% by chromosome analysis [3], is associated with a favorable prognosis, whereas the poorest prognostic indication by cytogenetics, deletion of the *TP53 *gene at 17p13, is seen in only 7% of FISH cases.

Microarray analysis has been well established for the genetic diagnosis of chromosomal disorders, and it was recently proposed that this technology be used as a first-tier test for a majority of children with clinical indications suggestive of a cytogenetic abnormality [5]. The use of arrays in the evaluation of hematologic malignancies is rapidly gaining use in response to the need for significantly greater molecular resolution to aid in diagnostic, prognostic and individualized decisions. In the case of pediatric acute lymphoblastic leukemia (ALL), microarrays have allowed for the identification of additional aberrations that are well below the limits of resolution for conventional cytogenetics and the determination of the prognostic significance for such findings [6-8]. Microarray studies using bacterial artificial chromosome (BAC)- or oligonucleotide (oligo)-based microarrays for the analysis of CLL have recently been described [9-11]. One study examining 174 CLL cases utilized a BAC array comprised of clones targeted to CLL-relevant loci and further defined abnormalities with higher-density oligonucleotide arrays and FISH [10]. For 89% of the cases, alterations were detected with the BAC arrays, and the limit of detection for clonal alterations was estimated to reside around 30%. An additional study showed that an appropriately designed microarray can detect clinically relevant findings that would be missed using FISH panels [9]. Recently, a few studies have demonstrated the use of single-nucleotide polymorphism (SNP) microarrays for the detection of chromosome aberrations [12,13]. In one study, a cross-platform comparison demonstrated the robustness of BAC arrays compared to SNP microarrays, which had relatively high levels of technical variation [12]. Accordingly, comparative genomic hybridization (CGH)-based arrays (e.g., BAC and oligo) are still likely the most appropriate platform for diagnostic use. Thus, microarrays offer value for diagnostic and prognostic determinations related to CLL, although precisely how such data will best be used in conjunction with established cytogenetic and FISH criteria has yet to be determined.

In this study we evaluated CLL cases using a whole-genome BAC microarray platform with increased coverage over regions of the genome associated with constitutional genetic syndromes and subtelomeric and pericentromeric regions. The results suggest that both CLL and CLL-predisposing microdeletions are readily detectable by microarray analysis.

## Methods

### Specimen ascertainment

Upon institutional review board approval (IRB 07245, 04187, 95124), we queried the City of Hope cytogenetic database to identify 33 patients with clinical indications of CLL and mantle cell lymphoma, who had residual material available for study.

The results of microarray analysis were compared to the patients' corresponding clinical, cytogenetic and pathological characteristics.

### Cytogenetics and FISH validation studies

Cytogenetic and FISH studies were performed using standard methods. The cytogenetics results were reviewed to confirm the karyotypic diagnosis, number of secondary karyotypic changes, and overall karyotype complexity. Whenever possible, at least 20 mitotic cells were analyzed, and the non-random cytogenetic aberrations were described according to ISCN (2009) [14]. Many of the aberrations observed by conventional cytogenetics were confirmed by FISH studies for follow-up minimal residual disease (MRD) testing using standard methods. Two-hundred cells were scored for interphase FISH (I-FISH).

### Microarray analyses

DNA was isolated from frozen buffy coat specimens using the EZ1 tissue kit and robot (Qiagen, Inc., Valencia, CA) per manufacturer's protocol. After isolation, DNA concentrations and quality were evaluated by spectrophotometry using the NanoDrop ND-1000 (NanoDrop Technologies, Wilmington, DE) and by agarose-gel electrophoresis. Microarray-based comparative genomic hybridization (aCGH) was performed using the SignatureChip^® ^Whole Genome™ (SignatureChipWG) BAC microarray using previously described methods [15]. Results were visualized using Signature's laboratory-developed computer software program Genoglyphix™ (http://www.signaturegenomics.com/genoglyphix.html). The nucleotide positions listed in SignatureChipWG v1.0.1 are based on the UCSC Genome Browser's March 2006 human reference sequence (hg18; NCBI Build 36.1). The aCGH results were described according to ISCN 2009 [14]. Normal (non-pathogenic) copy number variants (CNV) were not included in the aCGH results (Database of Genomic Variants, http://projects.tcag.ca/variation/.).

### Statistical analysis

The demographic and clinical covariates were compared using ANOVA for continuous variables and Fisher's exact test for categorical responses.

## Results

### Case selection for study

DNA was extracted from 33 bone marrow samples. Of these, 25 had a confirmed diagnosis of CL,L and five had mantle cell lymphoma. An additional three samples had insufficient DNA to perform microarray analysis. Of the 25 samples with CLL, 23 had prior chromosome analysis, and all had been examined by FISH for part or all of the CLL FISH panel (Table 1 and 2). Of the 25 cases evaluated, prior FISH evaluation showed that seven (28%) had deletion of the *ATM *gene, 12 (48%) had deletion at 13q14, seven (28%) had deletion of the *TP53 *gene, and five (20%) had trisomy 12. The distribution of abnormalities among the cases proved somewhat skewed toward the poorer prognostic indicators, given that trisomy 12 and *TP53 *deletions are typically less common (14% and 7%, respectively).

**Table 1:** Cases in which microarray analysis detected aberrations identified by chromosome analysis or FISH†

Case	Karyotype	FISH	Percent Abnormal by FISH	Array Results	Size
1	46,XY,del(11)(q21q23.3)[6]/46,XY[12]	nuc ish(CCND1,IGH@)x2 [217/217] nuc ish(CD82x2),(ATMx1)[138/200] nuc ish(CD82x2),(ATMx0)[29/200] nuc ish(pBR12,APAF1)x2[209/209] nuc ish(D13S319x1),(LAMP1x2)[129/200] nuc ish(TP53,ERBB2)x2[200/200]	0 69 14.5 0 64.5 0	del chr11:90634258-113746962 (11q14.3q23.2) del chr13:47624176-49406099 (13q14.2q14.3) del chr11:47479616-49590709 (11p11.2p11.12) del chr11:84811642-86481659 (11q14.1q14.2)	23.1 Mb 1.8 Mb 2.1 Mb 1.7 Mb

2	46,XX,der(3)del(3)(p13p21)inv(3)(p21p24),der(6)del(6)(q15q21)inv(6)(p21.3q23)[13]/46,XX[7]	nuc ish(CD82,ATM)x2[200/200] nuc ish(D13S319x1),(LAMP1x2)[157/200] IGH postive with 149/200 (74.5%) positive with loss of 5' signal in majority of cells.	0 78.5	del chr13:48903923-49406099 (13q14.3q14.3) del chr14:105267349-106339477 (14q32.33q32.33) dup chr1:912529-3454889 (1p36.33p36.32) del chr3:23938864-24184494 (3p24.2p24.2) del chr3:36967519-37301610 (3p22.2p22.2) del chr3:38387176-38681540 (3p22.2p22.2) del chr6:31007155-31476070 (6p21.33p21.33) del chr6:43741228-45692992 (6p21.1p12.3) del chr6:65600819-65840071 (6q12q12) del chr6:95941606-99824130 (6q16.1q16.2) del chr6:106626906-116596208 (6q21q22.1) dup chr13:113525857-113954547 (13q34q34) dup chr20:59074507-62317284 (20q13.33q13.33) del chr22:21330008-21570697 (22q11.22q11.22)	502 kb 1.1 Mb 2.5 Mb 246 kb 334 kb 294 kb 469 kb 2.0 Mb 239 kb 3.9 Mb 10.0 Mb 429 kb 3.2 Mb 241 kb IGL

5	42,X,-X,t(1;9)(q25;p13),add(5)(p13),der(8)t(8;13)(q13;q14),-9,der(12)t(12;?14) (q13;q11.2), -13,inv(14)(q11.2q32), psu dic(15;3)(p11.2;q27),der(17;21)(q10;q10),+der(17)t(17;21)(q10;q10)del(17)(q25)[15]/45,XX,t(1;10)(q21;q22),add(3)(q21),t(4;21)(q25;p11.2),t(6;19)(q21;p13.1),-9,del(9)(p13p24),inv(14)(q11.2q32), psu dic(15;3)(p11.2;q27),der(17;21)(q10;q10),+21[3] 43,XX,t(2;16)(q13;q22),del(7)(q22q32),der(8)t(8;13)(q13;q14),-9,add(9)(p13),der(12)t(12;?14)(q13;q11.2), inv(14)(q11.2q32),psu dic(15;3)(p11.2;q27),der(17;21)(q10;q10)del(17)(q25),+der(17;21)(q10;q10)add(21)(q22)[2]	nuc ish(TP53x1),(ERBB2x2)[139/200] nuc ish(TP53x1),(ERBB2x3)[9/200]	69.5 4.5	del chr17:0-21055067 (17p13.3p11.2) dup chr17:23775933-78654742 (17q11.2q25.3) dup chr1:912529-3548139 (1p36.33p36.32) del chr1:221094574-222727272 (1q41q42.12) del chr1:229351994-229691388 (1q42.2q42.2) del chr3:115404001-124892494 (3q13.31q21.1) dup chr3:127341829-129920493 (3q21.2q21.3) del chr3:133426459-133822407 (3q22.1q22.1) dup chr3:136891829-139071546 (3q22.3q23) del chr3:173687986-180776184 (3q26.3126.33) del chr5:1371093-12586305 (5p15.33p15.2) del chr8:77643686-79802767 (8q21.11q21.12) del chr8:115351618-115731259 (8q23.3q23.3) del chr9:188707-27863525 (9p24.3p21.2) del chr9:38261089-119714054 (9p13.1q33.1 ) dup chr12:2267492-5164844 (12p13.33p13.32) del chr12:84097500-132289534 (12q21.31q24.33) dup chr21:14429720-46912065 (21q11.2q22.3) del chr22:21044595-21570697 (22q11.22q11.22) del chrX:32390785-154763822 (Xp21.1q28)	21.1 Mb 54.9 Mb 2.6 Mb 1.6 Mb 339 kb 10 Mb 2.6 Mb 396 kb 2.2 Mb 7.1 Mb 11.2 Mb 2.2 Mb 380 kb 27.7 Mb 81.5 Mb 2.9 Mb 48.2 Mb Trisomy 526 kb 122 Mb

6	46,XY,t(4;13)(q31.3;q14),del(11)(q13q23)[13]/46,XY[7]	nuc ish(CD82x2),(ATMx1)[20/200] nuc ish(TP53,ERBB2)x2[199/200]	10.0 0	del chr13:46386690-49406099 (13q14.2q14.3) del chr11:49408635-49590709 (11p11.12p11.12) del chr3:166401814-166712788 (3q26.1q26.1) del chr11:79505241-113746962 (11q14.1q23.2) del chr14:105267349-106339477 (14q32.33q32.33) del chr22:21330008-21570697 (22q11.22q11.22)	3.0 Mb 182.1 kb 311 kb 34.2 Mb 1.1 Mb 241 kb

9	46,XY,del(11)(q13q23)[4]/ 46,XY[16]	nuc ish(D11Z1x2),(ATMx1)[12/211] nuc ish(DDIT3)x2[204/204] nuc ish(D13S319x1),(LAMP1x2)[10/200] nuc ish(CCND1,IGH@)x2[215/215] nuc ish(TP53x2),17cen(x2)[197/200]	5.7 0 5.0 0 0	del chr11:79505241-106371868 (11q14.1q22.3) del chr13:46386690-48065782 (13q14.2q14.2)	26.9 Mb 1.7 Mb

11§	45,XY,t(1;6)(p34.3;p23),der(14)?inv(14)(q22q32)t(8;14)(q21.2;q32.3),psu dic(20;17)(p12;p11.2)[2]/45,sl,der(9)t(8;9)(q22;q34)[3]/44,sdl1,-4,der(18)t(4;18)(q12;p11.2)[2]/ 88,sdl2x2,+9,+9,-der(9)t(8;9)(q22;q34)x2[cp5]/46,XY[8]	nuc ish(D11Z1,ATM)x4[74/213] nuc ish(DDIT3)x4[72/250] nuc ish(D13S319,LAMP1)x4[60/269] nuc ish(CCND1,IGH@)x4[75/200] nuc ish(TP53x1),17cen(x2)[55/200] nuc ish(TP53x2),17cen(x4)[67/200]	34.7 28.8 22.3 62.5 27.5 33.5	del chr4:12959459-96181870 (4p15.33q22.3) dup chr8:86064184-146236298 (8q21.2q24.3) del chr13:46386690-47967723 (13q14.2q14.2) del chr14:87377280-94457872 (14q31.3q32.13) del chr15:34882707-41330584 (15q14q15.2) del chr17:1-21055067 (17p13.3p11.2) del chr18:140284-5368662 (18p11.32p11.31) del chr20:9944-7553629 (20p13p12.3) dup chr20:16086026-19632379 (20p12.1p11.23) del chr22:21330008-21570697 (22q11.22q11.22)	83.2 Mb 60.2 Mb 1.6 Mb 7.1 Mb 6.5 Mb 21.1 Mb 5.23 Mb 7.5 Mb 3.6 Mb 240 kb IGL

12	47,XX,+12[2]/ 47,+12,t(2;14)(p13;q32.1)[5]/46,XX[15]	nuc ish(CD82,ATM)x2[262/263] nuc ish(pBR12,APAF1)x3 [91/200] nuc ish(D13S319,LAMP1)x2[203/204] nuc ish(CCND1,IGH@)x2[224/224] nuc ish(TP53,ERBB2)x2[200/200]	0 45.5 0 0 0	dup chr12:74345-132349534	Trisomy

13	46,XY,der(1)del(1)(p21p32)t(1;11;3)(q23;q23;q23),t(1;11;3)(q23;q23;q23)[4] Constitutional Cell Line: 46,XY[15]	nuc ish(CD82,ATM)x2[199/200] nuc ish(MLLx2)[209/209] nuc ish(Pbr12,APAF1)x2[200/200] nuc ish(D13S319,LAMP1)x2[210/211] nuc ish(TP53,ERBB2)x2[200/200]	0 0 0 0 0	del chr1:63608956-78245151 (1p31.3p31.1) del chr1:96795246-110397675 (1p21.3p13.3) del chr1:118792737-119283944 (1p12p12) dup chr17:9926840-10302007 (17p13.1p13.1)	14.6 Mb 13.6 Mb 491 kb 375 kb

14§	90 < 4n > XXYY,+3,add(9)(p13)x2,der(12)t(11;12)(q13;q24.3)x2,add(17)(p11.2)x2,-18,-18,-19[3]/ 90,sl,add(1)(p32),del(?15)(q11.2)[5]/87,sdl1,-add(1)(p32),-3,-6, del(14)(q24)[2]/46,XY[10]	nuc ish(CD82x4),(ATMx6)[112/219] nuc ish(pBR12,APAF1)x2[65/243] nuc ish(pBR12,APAF1)x4[178/243]nuc nuc nuc ish(D13S319,LAMP1)x4[135/208] nuc ish(CCND1x6)[13/220] nuc ish(CCND1x6),(IGH@x4)[127/220] nuc ish(TP53x2),(ERBB2x4)[139/200] nuc ish(CCND1,IGH@)x2[112/200] IGH break-apart showed 15.5% with normal pattern (2F), 6.2% with a 4F pattern, 55.9% with 2F/2R pattern, 22.4% with a 2F/1R (sdl 2) pattern.	51.1 73.3 64.9 5.9 57.7 69.5	dup chr11:66842920-134431368 (11q13.1q25) del chr17:1-22098241 (17p13.3p11.2) del chr1:912529-45151506 (1p36.33p34.1) dup chr2:44073-64590503 (2p25.3p14) dup chr3:46141-199230435 dup chr8:117699992-120401821 (8q23.3q24.1) del chr9:22407649-22847775 (9p21.3p21.3) dup chr10:3607230-9585338 (10p15.2p14) del chr12:122218562-132289534 (12q24.31q24.33) del chr14:105267349-106339477 (14q32.33q32.33) del chr15:22577151-100126412 del chr18:140284-46969866 (18p11.32q21.2) del chr19:211754-63770533	67.6 Mb 22.1 Mb 44.2 Mb 65.5 Mb Trisomy 2.7 Mb 440 kb 6.0 Mb 10.1 Mb 1.1 Mb Monosomy 46.8 Mb Monosomy

15	46,XY,del(11)(q21q23)[3]/ 46,XY[24]	nuc ish(D13S319,LAMP1)x2[99/200] nuc ish(CD82x2),(ATMx1)[14/200]	0 7.0	del chr11:90736134-113746962 (11q14.3q23.2) del chr5:141914285-145952287 (5q31.3q32) del chr16:21509120-21698983 (16p12.2p12.2)	23.0 Mb 4.0 Mb 190 kb

19	47,XY,+12[17]/46,XY[3]	nuc ish(CD82,ATM)x2[200/200] nuc ish(pBR12,APAF1)x3[172/200] nuc ish(D13S319,LAMP1)x2[216/216] nuc ish(CCND1,IGH@)x2[212/212] nuc ish(TP53,ERBB2)x2[200/200]	0 86.0 0 0 0	dup chr12:74345-132289534 del chr2:219234883-220344370 (2q35q35) del chr14:72506787-72834048 (14q24.2q24.2) del chr14:105267349-106339477 (14q32.33q32.33)	Trisomy 1.1 Mb 327 kb 1.1 Mb IGH

20	46,XY,add(11)(q23)[4]/46,sl,del(2)(q13q31),del(9)(p13p22),del(11)(q21q23)[3]/46,XY,inv(12)(p13q22)[6]/46,XY[7]	nuc ish(CD82x2),(ATMx1)[15/200] nuc ish(pBR12,APAF1)x2[200/200] nuc ish(D13S319x1),(LAMP1x2)[40/250] nuc ish(CCND1,IGH@)x2[205/205] nuc ish(TP53,ERBB2)x2[200/200] nuc ish(CDKN2Ax1)[70/200]	7.5 0 16.0 0 0 35.0	del chr13:45167078-76782310 (13q14.12q22.3) del chr9:14515097-22847775 (9p22.3p21.3) del chr2:95110968-151785533 (2q11.1q23.3) del chr3:46141-33554787 (3p26.3p22.3) del chr5:110467739-115587530 (5q22.1q23.1) del chr6:85275259-106796313 (6q14.3q21) del chr8:345060-111175147 (8p23.3q23.2) del chr11:100299542-118463179 (11q22.1q23.3) del chr14:105267349-105437150 (14q32.33q32.33) dup chr17:43041299-78654742 (17q21.32q25.3)	31.6 Mb 8.3 Mb 56.7 Mb 33.5 Mb 5.1 Mb 21.8 Mb 110.8 Mb 18.2 Mb 170 kb 35.6 Mb

21	46,XY,del(11)(q13q23)[4]/46,sl,add(2)(p23),add(3)(p25),add(8)(q24.1),add(12)(q22)[5]/45,sl,add(8)(q24.1),del(8)(p11.2p21),-13,der(22)t(13;22)(q14;p11.2)[6]/46,XY[8]	nuc ish(CCND1,IGH@)x2[211/211] nuc ish(D11Z1x2),(ATMx1)[163/207] nuc ish(DDIT3)x2[244/244] nuc ish(D13S319x1),(LAMP1x2)[27/200] nuc ish(D13S319x0),(LAMP1x2)[152/200] nuc ish(TP53x2),17cen(x2)[210/210]	0 78.7 0 13.5 76.0 0	del chr11:84811642-126916549 (11q14.1q24.2) del chr13:46660529-49406099 (13q14.2q14.3) del chr11:49408635-49590709 (11p11.12) dup chr2:60466431-61759445 (2p16.1p15) del chr6:55544176-78754776 (6p12.1q14.1) del chr8:2530350-5909600 (8p23.2p23.2) del chr8:39514037-39797061 (8p11.22p11.22) del chr8:13967960-17118468 (8p22p22) del chr8:115351618-116782788 (8q23.3q23.3) del chr11:49408635-49590709 (11p11.12p11.12)	42.1 Mb 2.75 Mb 182.1 kb 1.3 Mb 23.2 Mb 3.4 Mb 768 kb 3.2 Mb 1.4 Mb 182 kb

22	N.D.	nuc ish(D11Z1,ATM)x2[214/214] cells nuc ish(DDIT3)x3[18/205] nuc ish(D13S319x1),(LAMP1x2) [63/200] nuc ish(CCND1,IGH@)x2[214/215] nuc ish(TP53x2),17cen(x2)[199/200]	0 8.8 31.5 0 0	dup chr12:74345-132289534 del chr13:48903923-49406099 (13q14.3q14.3) del chr6:155195287-155551631 (6q25.2q25.2) del chr14:105267349-105437150 (14q32.33q32.33) del chr18:51018278-51378509(18q21.2q21.2)	Trisomy 502 kb 356 kb 170 Kb IGH 360 kb

23	46,XX[20]	nuc ish(D11Z1,ATM)x2[234/236] nuc ish(DDIT3)x2[222/222] nuc ish(D13S319,LAMP1)x2[203/204] nuc ish(CCND1,IGH@)x2[210/210] nuc ish(TP53x2),17cen(x2)[199/200]	0 0 0 0 0	del chr22:21330008-21570697 (22q11.22q11.22)	241 kb

† Nonspecific chromosome aberrations such as "add" or "del" were often redefined by the microarray results and are considered to have been detected.

§ Tetraploidy was not detected by microarray

N.D. = Not Done

Chr11 FISH [BAC 351G15 (ATM/11q22.3) and BAC 222Q13 (CD82/11p11.2) probes]

Chr12 FISH [12cen (pBR12) and APAF1/12q23 (BAC 210L7) probes]

Chr13 FISH [LSI D13S319 (13q14.3)/LSI LAMP1 (13q34), Vysis, Inc.]

Cyclin D1/IGH@ FISH [CCND1 (11q13)/IGH (14q32.3) probe, Vysis, Inc.]

Chr17 FISH [TP53 (17p13.1) BAC 199f11/ERBB2 (17q21.1) BAC 62n23 probes]

MLL/11q23 FISH [LSI MLL (11q23) probe, Vysis, Inc.]

MYC FISH LSI C-MYC (8q24.12-q24.13) probe Vysis, Inc.

### Specimens with concordance between microarray and FISH/chromosome analyses

**Figure 1:**
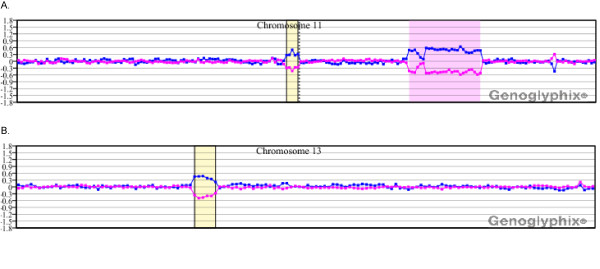
**Microarray results for case 1**. **(A) **Single-copy loss of probes at 11q14.1q11.23.2, approximately 28.9 Mb in size. This deletion interval includes the *ATM *locus. Probes are ordered on the x axis according to physical mapping positions, with the most distal p-arm probes on the left and the most distal q-arm probes on the right. The blue line represents the ratios for each clone from the first experiment (control/patient), and the pink line represents the ratios for each clone obtained from the second experiment in which the dyes have been reversed (patient/control). The yellow shaded region shows a deletion at 11p11.2p11.12 with a single BAC clone showing apparent homozygous deletion over the gene *PTPRJ*. Results are visualized using Genoglyphix (Signature Genomics). **(B) **Single-copy loss of probes at 13q14.2q14.3, approximately 1.8 Mb in size. This deletion interval includes the *RB1 *locus. Probes are arranged as in (A).

Fifteen of the 25 cases analyzed by microarray analysis showed detection of all copy number abnormalities identified by chromosome analysis and FISH (Table 1). For example, in case 1 cytogenetic analysis identified a single clonal abnormality [del(11)(q21q23.3] in six of 20 cells examined. FISH analysis detected deletion of a single allele of the *ATM *locus in 69% of the cells and biallelic deletion in 14.5%. Microarray analysis identified this deletion, defined the size (28.9 Mb) and refined the interval involved (11q14.1q23.2), which included the *ATM *locus (Figure 1A). Microarray analysis also identified a 1.8-Mb deletion at 13q14.2q14.3 that includes the *RB1 *locus, with the clinically significant *MIR16-1 *and *MIR15A *localized in the distal gap, which was not seen by conventional analysis but was identified by FISH in 64.5% of cells analyzed (Figure 1B).

### Additional complexity revealed by microarray analysis

**Figure 2:**
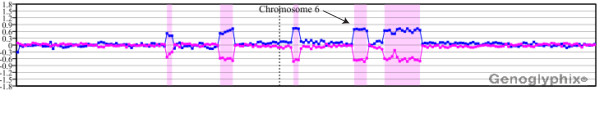
**Microarray characterization of a der(6)del(6)(q15q21)inv(6)(p21.3q23) in case 2**. In addition to a del(6)(q15q21) (arrow), single-copy losses were identified at each of the inversion breakpoints. Probes are arranged as in Figure 1A.

**Figure 3:**
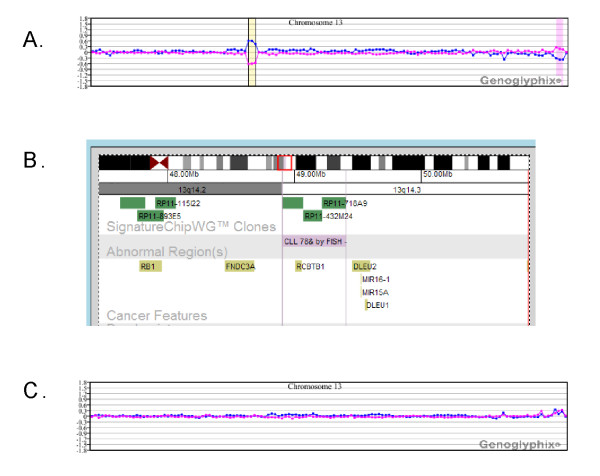
**Microarray results for cases with 13q14 deletions identified by FISH reveal insufficient coverage for the detection of small deletions**. **(A) **Microarray plot for case 2. For this case, the CLL FISH panel showed ~78.5% of cells with a 13q14 deletion. A deletion was detected by microarray analysis, but the extent of that deletion cannot be defined owing to insufficient coverage on the BAC array. **(B) **Insufficient coverage for the BAC array does not permit determination of the involvement of *MIR16-1 *and *MIR15A *in the deletion shown in part A. **(C) **Microarray plot for case 25. For this case, the CLL FISH panel showed ~20% of cells with a 13q14 deletion using a probe specific to *MIR16-1 *and *MIR15A*. Insufficient BAC coverage on this array is likely responsible for the failed detection of a small deletion. Plots are arranged as in Figure 1A.

In 15 of 25 (60%) cases, microarray analysis revealed additional complexity. For example, case 2 had both a der(3) and der(6) recognized by chromosome analysis, each of which was associated with an inversion event [der(3)del(3)(p13p21)inv(3)(p21p24), and der(6)del(6)(q15q21)inv(6)(p21.3q23]. The microarray results for chromosome 6 are shown in Figure 2. In addition to detection of the deletion, copy loss was identified at each of the inversion breakpoints. Gains at 1pter, 13qter and 20qter were also noted, as well as a terminal loss at 14q including the *IGH *locus. FISH analysis detected a 13q14 deletion in 78.5% of cells, and microarray analysis identified a deletion at 13q14.3; however, BAC clone coverage in the region is poor (Figure 3A, 3B). In case 15, microarray analysis detected an *ATM *deletion, the sole abnormality identified by both chromosomes and FISH.

In four cases with new abnormalities, the aberrations included a deletion within an interval defined as a putative CLL susceptibility locus. Case 1 showed a deletion at 11p11.2p11.12 with a single BAC clone showing apparent homozygous deletion over the gene *PTPRJ *(Figure 1A). Two additional cases (6 and 21) showed heterozygous single-BAC deletions at 11p11.12, approximately 1.3 Mb proximal to *PTPRJ*. The closest gene to this deletion is *FOLH1*, which resides in a gap between the deletion and the next most distal BAC contig. Case 2 had a 468.9-kb deletion at 6q21.33. Two additional cases, 18 and 20, showed large multi-megabase deletions that include the susceptibility region mapped to 5q22q23.

### Specimens with discordance between microarray and FISH/chromosome analyses

Ten specimens showed discordance between microarray results and prior FISH and/or chromosome analysis (Table 2). Three specimens, cases 3, 16 and 25, had normal microarray results (Table 2). Case 3 showed three cells out of 20 with trisomy 12 by chromosome analysis, but was only 2.5% positive for trisomy 12 by FISH following assessment with the full CLL FISH panel. Case 16 was a sample submitted for assessment of residual disease and had previously been shown to exhibit trisomy 12. However, the specimen analyzed in our study showed the abnormality to be present in one cell of 20 by chromosome analysis and 1.5% by FISH (Table 2).

**Table 2:** Cases in which microarray analysis did not detect aberrations identified by chromosome analysis or FISH

Case	Karyotype	FISH	Percent Abnormal by FISH	Array Results	Size
3	47,XY,+12[3]/46,XY[16]/92,XXYY[1]	nuc ish(CCND1,IGH@)x2 [202/202]	0	Arr(1-22)x2,(XY)x1	
		nuc ish(CD82,ATM)x2[199/200]	0		
		nuc ish(pBR12,APAF1)x3 [5/200]	2.5		
		nuc ish(D13S319,LAMP1)x2[204/204]	0		
		nuc ish(TP53,ERBB2)x2[200/200]	0		

4	46,XY,del(4)(p?15.2),del(11)(q21q23.3),add(14)(q32)[10]/46,XY[9]	nuc ish(ATMx1), (KAI1x2)[ 74/200]	37.0	del chr11:101900200-113746962 (11q22.2q23.2)	11.8 Mb
		nuc ish(ATMx1),(KAI1x1)[8/200]	4.0		
		nuc ish(D13S319x1),(LAMP1x1)[31/235]	13.2		
		nuc ish(D13S319x1),(LAMP1x2) [110/235]	46.8	del chr13:48903923-49406099 (13q14.3q14.3)	502 kb
		nuc ish(5'IGHVx1,3'IGHx1)[7/227]	3.1		
		nuc ish(5'IGHVx2,3'IGHx2)(5'IGHV sep 3'IGHx1)[35/227]	15.4		
		nuc ish(5'IGHVx1,3'IGHx2)(5'IGHV sep 3'IGHx1)[40/227]	17.6		
		nuc ish( (TP53,ERBB2)x2[250/250]	0		
		nuc ish(MYCx3)[ 116/200]	58.0	dup chr8:124436106-146015567 (8q21.13q24.3)	21.6 Mb
				del chr4:16516563-30324345 (4p15.32p15.1)	13.8 Mb
				del chr7:81021736-96580930 (7q21.11q21.3)	15.5 Mb

7	46,XY,t(14;19)(q32.3;q13.2)[9]/46,XY[11]	nuc ish(CD82,ATM)x2[199/200]	0		
		nuc ish(pBR12,APAF1)x2[199/200]	0		
		nuc ish(D13S319,LAMP1)x2[243/243]	0		
		nuc ish(CCND1,IGH@)x2[200/200]	0		
		nuc ish(TP53x1),17cen(x2)[10/202]	5.0		
				del chr14:105267349-106339477 (14q32.33q32.33)	170 kb
				del chr22:21330008-21570697 (22q11.22q11.22)	241 kb

8	46,XY,del(11)(q14q23)[4]/48,XY,+4,+inv(?18)(q21q23)[3]/46,XY[13]	nuc ish(CD82x2),(ATMx1)[21/200]	10.5		
		nuc ish(Pbr12,APAF1)x2[ 199/200]	0		
		nuc ish(D13S319x1),(LAMP1x2)[90/201]	44.8	del chr13:47759453-49406099(13q14.2q14.3)	1.6 Mb
		nuc ish(CCND1,IGH@)x2[200/200]	0		
		nuc ish(TP53,ERBB2)x2[199/200]	0		
				dup chr4:1-191273063	Trisomy
				dup chr18:1-76117153	Trisomy

10	47,XY,+12[9]/46,XY[11]	nuc ish(D11Z1x2),(ATMx3)[ 11/229]	4.8		
		nuc ish(DDIT3)x3[126/226]	55.8	up chr12:74345-132349534	Trisomy
		nuc ish(D13S319,LAMP1)x2[199/200]	0		
		nuc ish(CCND1,IGH@)x2[200/200]	0		
		nuc ish(TP53x1),17cen(x2)[11/213]	5.2		
				del chr22:21044595-21570697 (22q11.22q11.22)	526 kb

16	Previous Sideline: 47,XX,t(2;14)(p13;q32.1),+12[1]/46,XX[19]	nuc ish(pBR12,APAF1)x3[3/202]	1.5	Arr(1-22,X)x2	

17	Previous Sideline: 45,XY,del(3)(q21q27),-8,del(8)(p10),add(?9) (p13),del(11)(q13q23),del(13)(q14q22),add(17)(p10)[1]/46,XY[30]	nuc ish(CD82x2),(ATMx1)[6/200]	3.0	del chr11:84811642-124583747 (11q14.1q24.2)	39.8 Mb
		nuc ish(D13S319x1),(LAMP1x2)[44/243]	18.1	del chr13:46386690-57242066 (13q14.2q21.1)	10.9 Mb
		nuc ish(D13S319x0),(LAMP1x1 or 2)[33/243]	13.6		
		nuc ish(TP53x1),(ERBB2x2) [17/200]	8.5		
				dup chr2:44073-60812692 (2p25.3p16.1)	60.8 Mb
				del chr6:57972657-147822754 (6p11.2q24.3)	89.9 Mb
				del chr9:9839207-27863525 (9p23p21.2)	18.0 Mb

18	46,XY,t(3;11)(q21;p15)[,der(5)del(5)(q13q31)t(5;15)(q31;q13),r(?7)(p22q?22),der(15)t(5;15),-21[6]/46,XY[13]	nuc ish(CD82,ATM)x2[200/200]	0		
		nuc ish(pBR12,APAF1)x2[200 of 200]	0		
		nuc ish(D13S319,LAMP1)x1[27/200]	13.5		
		nuc ish(D13S319x1),(LAMP1x2)[82/200]	41.0		
		nuc ish(CCND1,IGH@)x2[228/228]	0		
		nuc ish(TP53,ERBB2)x2[195/200]	2.5		
				del chr3:66524310-71438751 (3p14.1p14.1)	4.9 Mb
				del chr5:67408207-67790791 (5q13.1q13.1)	382 kb
				del chr5:76836011-180616147 (5q14.1q35.3)	104 Mb
				del chr7:2327342-54294538 (7p22.3p11.2)	54.2 Mb
				del chr7:81021736-96580930 (7q21.11q21.3)	15.6 Mb
				del chr7:102955780-158788150 (7q22.1q36.3)	55.8 Mb
				del chr21:41514980-46912065 (21q22.3q22.3)	5.4 Mb

24	46,XY[20]	nuc ish(D11Z1,ATM)x2[213/213]	0		
		nuc ish(DDIT3)x2[229/229]	0		
		nuc ish(D13S319,LAMP1)x1[15/203]	7.4	del chr13:48903923-49406099 (13q14.3q14.3)	502.2 kb
		nuc ish(D13S319x1),(LAMP1x2)[155/203]	76.3		
		nuc ish(CCND1,IGH@)x2[223/223]	0		
		nuc ish(TP53x2),17cen(x2)[199/200]	0		

25	N.D.	uc ish(D11Z1,ATM)x2[212/213]	0	Arr(1-22,X)x2	
		nuc ish(DDIT3)x2[204/204]	0		
		nuc ish(D13S319x1),(LAMP1x2)[13/217]	6.0		
		nuc ish(D13S319x0),(LAMP1x2)[30/217]	13.8		
		nuc ish(D13S319x0),(LAMP1x2)[30/217]	0		
		nuc ish(TP53x2),17cen(x2)[199/200]	0		

**Figure 4:**
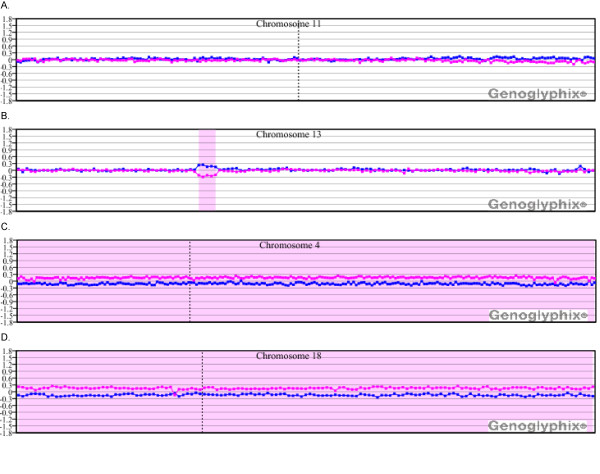
**Microarray results for case 8 showing discordance between microarray and FISH**. **(A) **Microarray analysis did not detect the deletion at *ATM *that was identified in 10.5% of cells by FISH. **(B) **Microarray detected the 13q14 deletion that was present in 44.8% by FISH. Microarray analysis also detected **(C) **trisomy 4 and **(D) **trisomy 18. Probes are arranged as in Figure 1A.

**Figure 5:**
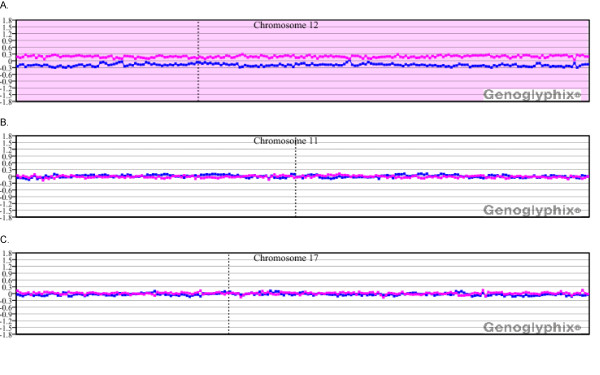
**Microarray results for case 10 showing discordance between FISH and microarray results**. **(A) **Microarray plot showing trisomy 12, which was in 55.8% of cells by FISH. **(B, C) **Normal microarray plots for (B) chromosome 11 and (C) chromosome 17. Probes are arranged as in Figure 1A.

In several cases, microarray analysis failed to detect aberrations that were identified by prior FISH and/or chromosome analysis. For most of these cases abnormalities detected by FISH and/or chromosome analysis that were present in at least 10% of cells were detected by microarray, whereas values below 10% were not likely to be detected (Table 2). However, there were exceptions. Cases 4, 8, 10, 17 and 24 all yielded mixed results, with detection of one or more abnormalities and a concurrent failure to detect others. Microarray analysis of cases 10, 17 and 24 confirmed all aberrations present in more than 10% of the cells by FISH but failed to detect most that scored below 10% on FISH (see Table 2). In case 8, FISH identified deletions at *ATM *(10.5%) and 13q14 (44.8%), but the *ATM *alteration was not confirmed by array (Figure 4A, B). For comparison, chromosome analysis of that same case had also identified the *ATM *deletion, as well as trisomy for chromosomes 4 and 18 (3/20 cells). Although microarray analysis failed to detect the *ATM *deletion, trisomy 4 and 18 were both detected (Figure 4C, D). Case 10 exhibited trisomy 12 in 55.8% of cells by FISH and deletions of both *ATM *and *TP53 *at levels below 10%. Microarray analysis was only able to detect the trisomy (Table 2, Figure 5). For case 4, FISH-identified deletions of *ATM*, 13q14 and *IGH *all exceeded 10%, and all were detected by array. However, monosomies for chromosomes 11 (4%), 13 (13.2%) and 14 (3.1%) that were detected by FISH were not detected by array. In contrast, for case 9, low-level deletions for both *ATM *(5.7%) and 13q14 (5.0%) were detected by microarray.

Chromosome analysis was not performed on case 25; however, the CLL FISH panel showed ~20% of cells with a 13q14 deletion, the majority showing biallelic loss. The microarray results were normal, including the BAC clones covering 13q14 (Figure 3C). Given the high frequency of biallelic loss detected by FISH, it is surprising that the microarray failed to detect this abnormality. For case 7, chromosome analysis identified t(14;19)(q32.3;q13.2) in nine of 20 cells evaluated, and FISH analysis with the CLL panel detected a *TP53 *gene deletion in only 5% of cells. Microarray analysis was normal with the exception of small gene-specific deletions for *IGL *and *IGH*. The *IGH *deletion may be specifically related to t(14;19)(q32.3;q13.2) translocation. The translocation breakpoint on chromosome 19, representing the *BCL3 *gene, is not well covered on the microarray, and the detection of a breakpoint-specific alteration would not be expected. The fact that the *TP53 *gene deletion seen by FISH was not detected is not surprising, because 5% is below the anticipated limits of microarray analysis.

For case 18, microarray results detected all abnormalities detected by chromosome analysis but none of the aberrations detected by FISH. Deletion 13q14 (41%), monosomy 13 (13.5%) and *TP53 *deletion (2.5%) were all undetected. One additional case, 23, had both normal cytogenetics and normal results for the full CLL FISH panel. The case was evaluated to determine whether there were abnormalities below the resolution of routine assays. The only alteration detected was a small deletion involving the *IGL *locus.

### Detection of microarray abnormalities within a mosaic tetraploid background

**Figure 6:**
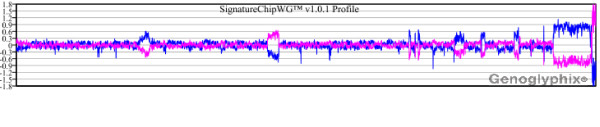
**Microarray characterization of a sample compromised by mosaic tetraploidy (case 11)**. Despite the mosaic tetraploidy, deletions and duplications of multiple chromosomal regions were identified. These are outlined in greater detail in Table 1. A whole-genome view is displayed with chromosome 1 on the left and the sex chromosomes on the right. The sex chromosome pattern is consistent with a male specimen run against a female control. Probes are arranged as in Figure 1A.

In two cases (11 and 14), microarray analysis identified aberrations in cells shown by chromosome analysis to represent mosaic tetraploidy. For case 11 (25% tetraploidy), aberrations identified by conventional chromosome analysis, which were seen in both diploid cells and also in tetraploid cells following reduplication, were identified by microarray (Figure 6). In contrast to case 11, cytogenetic aberrations seen on cytogenetic analysis in case 14 (50% tetraploidy) were confined to the tetraploid cell population. The abnormalities were not present in the diploid cell population. However, the abnormalities associated with this specimen were also detected by aCGH with a total of 13 aberrations identified. As expected, the mosaic tetraploidy was not detectable in either case.

## Discussion

Several recent studies have suggested that microarrays are likely to improve diagnostic and prognostic determinations related to CLL, although it has not been determined how such data will best be used in conjunction with established cytogenetic and FISH criteria. In this study we evaluated 25 CLL samples by microarray analysis. All had prior FISH analysis for one or more probes, and 23 cases had prior chromosome analysis (Tables 1 and 2). Excluding three cases with normal array results, 22 cases had abnormal microarray findings. In 15 cases (60%), microarray analysis identified new abnormalities not recognized by cytogenetic analysis and FISH. These results demonstrate the clinical utility of microarray analysis for the identification of cytogenetic aberrations related to CLL. Of course, one should consider the possibility of an underlying concurrent condition such as myelodysplastic syndromes (MDS). However, for the majority of the cases in this study no morphologic evidence of MDS was seen, and most had extensive bone marrow or peripheral blood involvement by CLL, so genetic changes are likely CLL related.

With several exceptions, microarray analysis was able to identify abnormalities present in as few as 10% of cells analyzed by chromosome analysis or FISH. The sensitivity of detection for known abnormalities was improved over the 30% that has been previously reported for the analysis of CLL cases by others [10]. While prior experience with constitutional clinical cases using this array suggests an average detection of approximately 20% mosaicism [16], in the current study that limit of detection was closer to 10%, albeit with certain exceptions (Table 1 and 2).

Several cases had minimal or normal microarray results that were surprising and may reflect inadequate array clone coverage. Failure to detect the chromosome 13 alterations, particularly the 13q14 deletion, is unexpected based on the limits defined by other cases. Microarray coverage may have been inadequate to confirm a small genomic deletion. The FISH probe routinely used in these studies maps in a gap telomeric to the BAC clone coverage near the *RB1 *locus. Smaller deletions in CLL cases within this gap that do not include the *RB1 *locus, but do include the clinically relevant *MIR16-1 *and *MIR15A *genes, have been described [10]. For case 2, aCGH revealed deletion of a contig of three BAC clones that reside between the *RB1 *gene and *MIR16-1 *and *MIR15A*. Coverage was sufficient to exclude the inclusion of the *RB1 *in the deletion, but as with the previous case, this BAC array lacked sufficient resolution to identify *MIR16-1 *and *MIR15A *deletion as defined by FISH (Figure 3A-C). These results suggest the need for higher density and specifically targeted coverage for these critical regions of interest.

A similar circumstance was seen for *ATM *deletion detection, which suggests that inadequate coverage in addition to low-level representation provide a likely explanation for the failure to detect these deletions when seen by FISH. Although the Vysis FISH probe is positioned directly over the *ATM *locus, the nearest BAC contig to this locus on our constitutional array is 1.2 Mb proximal. Finally, although our microarray has direct coverage over *TP53*, those cases in which detection was missed all had representation in less than 10% of the nuclei by FISH.

BAC-based aCGH is not designed to optimally detect differences in ploidy owing to inherent limitations in the technology. Because equal quantities of diploid (control) and triploid or tetraploid (specimen) DNA are compared, in the absence of any additional abnormalities, the total number of chromosomes represented in both the specimen and control samples will be equivalent, precluding the detection of tetraploidy. For constitutional cases it has been shown that careful examination of the sex chromosome ratios between the patient sample and the control can facilitate the detection of triploidy, but not tetraploidy, if the specimen has been run against an opposite-sex control [17]. The detection of triploidy or tetraploidy in oncology cases constitutes an important challenge that is likely to suffer from the same limitations described and may be further complicated by the potential mosaic nature of the ploidy. Furthermore, additional copy changes present within the mosaic triploid/tetraploid cell population might be masked by the dilution that effectively occurs in matching DNA quantity to that of a control. In the case of tetraploidy, if a specific alteration in question is present at two copies per cell as a result of chromosome replication without division (i.e., +12 × 2 in 4n cells), then the ratio of one abnormal chromosome per homologous pair is preserved. However, if that same alteration arose as a single event within a tetraploid cell, then it is present on only one of the four homologues (i.e., +12 × 1 in 4n cells). The log_2 _ratio for the latter specimen would be approximately half that seen for the former.

While the detection of ploidy remains an issue beyond the limits of the BAC microarray technologies, our results demonstrate that microarray analysis can detect additional aberrations in a known mosaic tetraploid background. Two male cases in the current study, case 11 and case 14, showed mosaic tetrasomy by both FISH and conventional cytogenetics. For case 11, tetrasomy was seen in 25-35% of the cells, whereas for case 14 such cells constituted approximately 50% of cells. For case 11, all of the aberrations identified by conventional chromosome analysis were seen in near-diploid cells. In contrast, the cytogenetic aberrations in case 14 identified by conventional chromosome analysis were confined to the tetraploid cells, with all diploid cells exhibiting a normal karyotype. As expected, aCGH was not able to detect the presence of tetraploidy in either specimen. However, for both samples, it was possible to detect those aberrations previously noted by cytogenetics and/or FISH and identify additional abnormalities (Table 1, Figure 6).

Epidemiological studies suggest the existence of susceptibility loci for CLL, with large numbers of families showing disease clustering, including large pedigrees with inheritance consistent with dominantly acting alleles [18-22]. In at least one study, risk for CLL was estimated to be increased seven-fold in first-degree relatives of CLL patients, while the risk for Hodgkin and non-Hodgkin lymphoma was elevated two-fold in the relatives of CLL patients [23,24]. Multipoint linkage analyses using an 11,560-SNP array identified the highest linkage disequilibrium (LD) on chromosome 11p11, with the same genomic interval demonstrating the highest multipoint heterogeneity LOD (HLOD) score [25]. Potential candidate genes mapping to this interval include *PTPRJ*, *MADD *and *DDB2*. HLOD values also suggested possible loci at 5q22q23, 6p22, 10q25, and 14q32, all distinct from those regions commonly evaluated in CLL. In another study analyzing 206 families with a history of CLL, the most significant linkage was established at chromosome 2q21.2, with a suggestion of additional potential loci at 6p22.1 and 18q21.1 [26]. One report described the refined mapping of a chromosome 13 CLL predisposition locus to a 3.7-Mb interval at 13q21.33q22.2; however, sequencing of 13 genes in the region identified no germline mutations. Indeed, the specific genes within any of these intervals that contribute to CLL predisposition have yet to be elucidated.

The 11p11.2p11.12 deletion observed in case 1 in our study is well delineated by sufficient BAC coverage on the distal boundary to conclude that *PTPRJ*, but not *MADD *and *DDB2*, is deleted. *PTPRJ *encodes a type J protein tyrosine phosphatase receptor that regulates cellular proliferation and differentiation. The value for the log_2 _ratio for this interval is -0.306, consistent with heterozygous deletion in all cells. Although we cannot determine whether the deletion at 11p11.2p11.12 was inherited or acquired, owing to the blinded nature of this study, detection of the deletion of *PTPRJ *in a case of CLL is intriguing. Two additional cases had single-BAC deletions at 11p11.12, approximately 1.3 Mb from the *PTPRJ *gene. In contrast, case 2 showed a 468.9-kb deletion at 6p21.33 that includes the *HLA-C *and *HLA-B *loci, which have been implicated in CLL predisposition in the previous mapping studies [25,26]. Two additional cases (18 and 20) showed deletions that include a previously identified susceptibility region at 5q22q23, but these deletions were large multi-megabase alterations not likely to have been inherited. The identification of alterations at putative CLL predisposition loci in four out of 25 samples represents an exceptionally high detection rate for familial cases. However, all specimens analyzed in this study were from patients referred to a tertiary care center. Thus, there may be a bias in representation toward the more severely affected end of the CLL clinical spectrum, which may include a greater proportion of inherited cases.

Finally, and not surprisingly, analysis of 25 cases with this array platform revealed at least one abnormality that is not related to cancer but is clinically relevant. Microarray analysis revealed a heterozygous loss at 16p12.2, likely to be constitutional in nature. Patients with such deletions have been seen previously in the laboratory and are carriers for autosomal recessive hearing loss type 22 (DFNB22) caused by an alteration in the *OTOA *gene [27,28].

## Conclusion

We have performed microarray analysis of 25 previously diagnosed CLL cases using a microarray platform optimized for the assessment of prenatal and postnatal inherited disorders. The results demonstrate exceptionally good resolution with the detection of low-level mosaicism (~10%), most likely attributable to the robust performance of the platform design. Moreover, 15 of the cases (60%) revealed additional complexity or new abnormalities not recognized during traditional analysis, illustrating the potential of microarrays to detect alterations with relevance and susceptibility to the acquired neoplastic disease, and inherited alterations with relevance to constitutional disorders. These results are consistent with the notion that CLL could be incidentally diagnosed during the course of constitutional microarray analysis of an adult patient, the population with greatest risk of presenting with this fairly prevalent and under-diagnosed disorder. Importantly, discordant results between cytogenetic analysis/FISH and microarray analysis in 10/25 (40%) of cases in this study demonstrate the need for a broad but targeted microarray optimized for the detection of cancer-related abnormalities.

## Competing interests

RS, LM, BCB and LGS are employees of Signature Genomics, a subsidiary of PerkinElmer.

## Authors' contributions

RS was the principal investigator, wrote the paper and takes primary responsibility for the paper. MD and SJF provided the test samples and clinical information. KG performed the pathology review. VB performed the laboratory work for this study. DDS participated in the statistical analysis. MLS, BCB, and LGS coordinated the research. LM was involved in the discussions. All authors have read and approved the final manuscript.
